# Nationwide Survey of Vector-Borne Diseases in Rodents and Mites in Korea: *Anaplasma*, *Ehrlichia*, and *Rickettsia*

**DOI:** 10.3390/ani14202950

**Published:** 2024-10-13

**Authors:** Beoul Kim, You-Jeong Lee, Dongmi Kwak, Min-Goo Seo

**Affiliations:** College of Veterinary Medicine & Institute for Veterinary Biomedical Science, Kyungpook National University, 80 Daehak-ro, Buk-gu, Daegu 41566, Republic of Korea; kbjjhnm@naver.com (B.K.); wowgirlsgood@naver.com (Y.-J.L.); dmkwak@knu.ac.kr (D.K.)

**Keywords:** wild rodents, mites, *Anaplasma*, *Ehrlichia*, *Rickettsia*

## Abstract

**Simple Summary:**

Rodents are known to carry a variety of pathogens that can cause diseases in humans, especially those transmitted through small parasites like mites. This study investigated the presence of three specific bacteria, *Anaplasma*, *Ehrlichia*, and *Rickettsia*, in rodents and their mites across Korea in 2022 and 2023. We found that 10.3% of the 835 rodents were infected with *Anaplasma phagocytophilum*, 0.5% with *Ehrlichia muris*, 0.2% with *Ehrlichia ruminantium*, and 2.9% with *Rickettsia raoultii*. In addition, 8.8% of 7971 mites tested positive for *Anaplasma*, and lower percentages of the mites tested positive for the other bacteria. Importantly, this is the first time these bacteria have been detected in rodents and mites in Korea, and in some cases, globally. Our findings highlight the need for ongoing monitoring and better systems to track these diseases to prevent future outbreaks, especially as climate and environmental changes may increase their spread. This information is vital for public health awareness and protection from vector-borne diseases.

**Abstract:**

Rodents are reservoirs for zoonotic pathogens, making it essential to study both rodents and their ectoparasites. In 2022 and 2023, we investigated the spatial distribution of rodents and their mites across Korea, focusing on three vector-borne diseases (VBDs): *Anaplasma*, *Ehrlichia*, and *Rickettsia*. A total of 835 wild rodents were collected from 16 locations, each consisting of five distinct environmental settings (mountains, waterways, reservoirs, fields, and paddy fields), with 20 traps per setting, totaling 100 Sherman live folding traps per site. Each rodent was identified using a taxonomic key, and post-mortem examinations led to the collection of 7971 mites (498 pools), followed by PCR analysis. Among the rodents, *Anaplasma phagocytophilum* was detected in 10.3%, *Ehrlichia muris* in 0.5%, *Ehrlichia ruminantium* in 0.2%, and *Rickettsia raoultii* in 2.9%. In mites, *A. phagocytophilum* was found in 8.8%, *E. muris* in 0.2%, *R. raoultii* in 0.2%, *R. endosymbiont* in 1.6%, and *R. australis* in 1.2%. This study marks the first detection of *E. muris* and *R. raoultii* in Korean rodents and the first global discovery of *E. ruminantium* in rodents. The detection of multiple pathogens in mites worldwide highlights the importance of continuous VBD monitoring to mitigate public health risks.

## 1. Introduction

There is growing recognition of the impact that changing climates, environments, and habitats have on the risk of infectious zoonotic diseases, highlighting their significance [1]. Vector-borne diseases (VBDs) are infections transmitted from animals to humans via vectors, causing over 1 billion infections and 1 million deaths annually [2]. In Korea, data from the Korea Disease Control and Prevention Agency (KDCA) over the last decade indicate a steady rise in the incidence of VBDs among the Korean population [3]. Despite the KDCA’s continuous nationwide surveys on mite distribution, especially concerning diseases such as scrub typhus, there remains a significant gap in surveillance and research on other mite-borne diseases, unlike the attention given to tick-borne diseases [4]. Furthermore, rodents harbor a range of zoonotic pathogens, making it essential to investigate both the captured rodents and their ectoparasites simultaneously to assess their roles in disease transmission.

Rickettsial diseases are emerging and illnesses caused by obligate intracellular bacteria transmitted by arthropods are resurfacing, affecting humans and animals globally. Rickettsiales, including *Anaplasma*, *Ehrlichia*, and *Rickettsia*, are transmitted to companion, domesticated, and wild animals via bites from infected fleas, lice, ticks, or mites [5]. Numerous cases have reported the detection of VBDs, such as *Anaplasma phagocytophilum* in ticks and rodents in China [6], *Ehrlichia muris* in ticks and rodents in the United States [7], and in rodents in Slovakia [8], *Rickettsia raoultii* in rodents in Kazakhstan [9], and *Rickettsia endosymbionts* in rodents in Slovakia [10].

Anaplasmosis and ehrlichiosis are zoonotic diseases caused by Gram-negative, obligate intracellular bacteria from the family *Anaplasmataceae* [11]. *A. phagocytophilum* is the causative agent of human granulocytic anaplasmosis (HGA), which has been detected in various tick species [12] and rodents in Korea [13], and the first human case of HGA in Korea was reported in 2013 [14]. Ehrlichiosis is an emerging zoonotic disease of significant clinical importance. Human ehrlichiosis is primarily caused by *Ehrlichia chaffeensis*, which infects monocytes, and less commonly by *Ehrlichia ewingii*, which infects granulocytes, in the United States [15]. *E. ewingii* and *E. chaffeensis* are transmitted to humans through the bite of an infected *Amblyomma americanum* tick [16]. Additionally, *Ehrlichia* species have been detected in ticks collected from humans in Korea [17].

Species of *Rickettsia* are emerging or reemerging pathogens relevant to public health, especially since tick-borne rickettsioses result from obligate intracellular bacteria within the spotted fever group of the genus *Rickettsia* [18]. In recent decades, many species of Rickettsiae transmitted by ticks, once considered nonpathogenic, have been identified as human pathogens [19]. Among the various species of *Rickettsia*, *R. raoultii* causes tick-borne lymphadenitis in several European countries [20], and it has also been detected in ticks collected from patients bitten by ticks in Korea [21].

Previous studies indicate that VBDs continuously occur in ticks and rodents worldwide, but research on mites remains limited. Furthermore, investigation into VBDs, particularly mite-borne zoonotic diseases among rodent and mite populations in Korea, are scarce. Therefore, this research aims to bridge this gap by conducting comprehensive comparisons and analyses of infection rates, forecasting potential disease outbreaks, and implementing a robust surveillance system for rodents and mites in Korea. By enhancing our understanding of the prevalence and distribution of these pathogens, this study seeks to provide valuable insights into the epidemiology of VBDs in Korea.

## 2. Materials and Methods

### 2.1. Ethical Approval

The animal experimentation protocol was approved and reviewed in compliance with the scientific care standards and ethical procedures set forth by the Institutional Animal Care and Use Committee (IACUC) of the Korea Centers for Disease Control and Prevention (KCDC-093-18). The mite and rodent specimens used in this study were supplied by the KDCA. Additionally, this research underwent a review and obtained approval in accordance with the ethical protocols and scientific care guidelines of the IACUC at Kyungpook National University (KNU 2022-0441).

### 2.2. Surveillance of Rodents

In partnership with the KDCA-operated Regional Center for Vector Surveillance against Climate Change, rodents were trapped at 16 sites across the country using rodent traps. Rodent-collecting areas were classified into northern, central, southern, and Jeju Island regions. In the northern area, samples were gathered from Hwaseong, Paju, Yeoju, Cheorwon, and Gangneung. The central area included Cheongju, Boryeong, and Yesan. The southern area comprised Jeongeup, Boseong, Jinan, Geoje, Hapcheon, Gimcheon, and Yeongdeok. Samples were gathered in Seogwipo on Jeju Island. Collection activities were conducted six times at each site during the spring and autumn of 2022, as well as during the spring of 2023. Each collection site included five distinct environmental locations (mountains, waterways, reservoirs, fields, and paddy fields), where a total of 100 Sherman live folding traps were set up. Traps baited with peanut butter-spread cookies were placed at intervals of 3–5 m within each area, totaling 20 traps per environmental location. The traps were arranged prior to sunset and retrieved the following morning. After transporting the collected traps to the laboratory, the rodents were swiftly euthanized using CO_2_ gas, and each rodent species was identified using a taxonomic key [22]. Liver tissue samples were then gathered and preserved at −70 °C for further analysis.

### 2.3. Surveillance of Mites

To gather external ectoparasites, such as mites, from each euthanized rodent, the rodent was hung upside down by the tail on a rod above a beaker filled with water, positioned inside a Petri dish. This setup enabled the collection of external parasites as they dislodged over a 24 h incubation period onto the surface of the Petri dish. The mites were recovered from the water surface with a fine brush. The gathered mites were subsequently preserved in 70% ethanol for further analysis. At the KDCA, approximately half of the mites collected from each rodent were provided for this study to detect VBDs, while the remaining mites were preserved as a biological resource for long-term use. The mites collected from each rodent were visually counted one by one under an optical microscope, and from the large number of mites, a random sample of 1-to-30 mites was selected for pooling experiments. Morphological species identification of the mites, which is typically performed by mounting individual mites on glass slides using polyvinyl alcohol mounting medium and examining them under an optical microscope with morphological keys, was not conducted in this study. This decision was made to streamline the process for DNA extraction and PCR analysis.

### 2.4. Molecular Detection of VBDs

To isolate DNA, we homogenized both mite pools (consisting of 1–30 mites per rodent) and rodent liver tissues using the Precellys CK28-R Lysing kit (a bead tube designed for hard tissue homogenization, Bertin Technologies, Bretonneus, France) and the Precellys evolution Homogenizer (Bertin Technologies). Following homogenization, genomic DNA extraction was performed using the DNeasy Blood & Tissue Kit (Qiagen, Melbourne, Australia) according to the manufacturer’s instructions. The 16S rRNA sequence of Rickettsiales was detected via PCR using the commercial AccuPower Rickettsiales 3-Plex PCR Kit (Bioneer). PCR was performed in a 20 μL reaction volume containing 5 μL of template DNA, with an initial denaturation at 95 °C for 15 min, followed by 40 cycles of denaturation at 95 °C for 10 s, and annealing and extension at 58 °C for 45 s. A final extension was performed at 72 °C for 5 min. PCR products were visualized using agarose gel electrophoresis and stained with ethidium bromide.

### 2.5. DNA Sequencing and Phylogenetic Investigation

The PCR-positive products were purified using ExoSAP-IT™ (PCR product cleanup reagent, Thermo Fisher Scientific, Waltham, MA, USA) and sent to Macrogen (Seoul, Republic of Korea) for sequencing, along with the primers. Nucleotide sequences of these confirmed pathogens were obtained through sequencing. Comparative analysis was subsequently conducted by aligning these sequences with entries in the NCBI’s GenBank database for molecular genetic examination. The multiple sequence alignment tool CLUSTAL Omega (v. 1.2.1, Bioweb, Ferndale, WA, USA) was employed to align and analyze the obtained sequences alongside those previously documented in GenBank. Following identification using BioEdit (v. 7.2.5, BioEdit, Manchester, UK), redundant sequences were removed. Phylogenetic analysis was performed using MEGA (v. 6.0, Mega Software Solutions, Madhurawadha, India) [23], employing the Kimura two-parameter distance model [24] and the maximum likelihood method.

### 2.6. Statistical Examination

Statistical analysis was performed using GraphPad Prism version 5.04 (GraphPad Software Inc., La Jolla, CA, USA). Pearson’s chi-square test was used to analyze contingency tables with more than two variables (e.g., regions and rodent species), while Fisher’s exact test was applied to 2 × 2 tables (e.g., seasons). Statistical significance was set at a *p*-value of ≤0.05.

## 3. Results

### 3.1. Rodent Identification

A total of 835 rodents were captured across 16 locations. After classification, they were grouped into ten species: *Apodemus agrarius* (82.9%), *Apodemus peninsulae* (0.6%), *Craseomys regulus* (1.6%), *Craseomys rufocanus* (0.1%), *Crocidura lasiura dobson* (0.1%), *Crocidura* spp. (10.3%), *Micromys minutus* (3.0%), *Microtus fortis* (0.5%), *Mogera robusta* (0.1%), and *Myodes regulus* (0.7%) (Table 1). The predominant species among the gathered rodents was *Ap. agrarius*, which also demonstrated the highest infection rate for VBDs at 17.2% (119/693). Of the 835 rodents captured across diverse habitats, the distribution was as follows: mountains (20.7%), waterways (25.1%), reservoirs (20.8%), fields (19.4%), and paddy fields (13.9%).

### 3.2. Presence of VBDs in Rodents

When examining the agent positivity rates among rodent species, 10.3% tested positive for *A. phagocytophilum*, 2.8% for *E. muris*, 0.2% for *E. ruminantium*, and 2.9% for *R. raoultii*. No detections were made for *R. endosymbiont* and *R. australis*. Statistical analysis using Pearson’s chi-square test was conducted for the rodent data; however, none of the results were statistically significant (*A. phagocytophilum*: *p* = 0.0635; *E. muris*: *p* = 0.8475; *E. ruminantium*: *p* = 1; *R. raoultii*: *p* = 0.9938) (Table 1). No statistical values were obtained for the mites.

For regional comparisons (Table 2), Pearson’s chi-square test revealed statistically significant results for some pathogens (*A. phagocytophilum*: *p* < 0.0001; *E. muris*: *p* < 0.0001; *E. ruminantium*: *p* = 0.3049; *R. raoultii*: *p* < 0.0001). Similarly, seasonal analysis (Table 2) using Fisher’s exact test showed statistically significant results for some pathogens (*A. phagocytophilum*: *p* < 0.0001; *E. muris*: *p* < 0.0001; *E. ruminantium*: *p* = 0.0433; *R. raoultii*: *p* < 0.0001).

### 3.3. Presence of VBDs in Mites

Across 16 distinct locates, a combined total of 7971 mites (comprising 498 pools) were gathered from 835 captured rodents (Table 2). Among them, *A. phagocytophilum* tested positive in 8.8%, while *E. muris* and *R. raoultii* each tested positive in 0.2%. *R. endosymbiont* tested positive in 1.6%, and *R. australis* in 1.2%. However, *E. ruminantium* was not detected.

For regional comparisons (Table 2), Pearson’s chi-square test revealed statistically significant results for some pathogens (*A. phagocytophilum*: *p* = 0.0014; *E. muris*: *p* = 0.4812; *R. raoultii*: *p* = 0.9333; *R. endosymbiont*: *p* = 0.0043; *R. australis*: *p* = 0. 3917). Similarly, seasonal analysis (Table 2) using Fisher’s exact test showed statistically significant results for some pathogens (*A. phagocytophilum*: *p* < 0.0001; *E. muris*: *p* = 0.4832; *R. raoultii*: *p* = 0.4832; *R. endosymbiont*: *p* = 0. 0464; *R. australis*: *p* = 0. 0849).

### 3.4. Genetic and Phylogenetic Analyses

**Figure 1:**
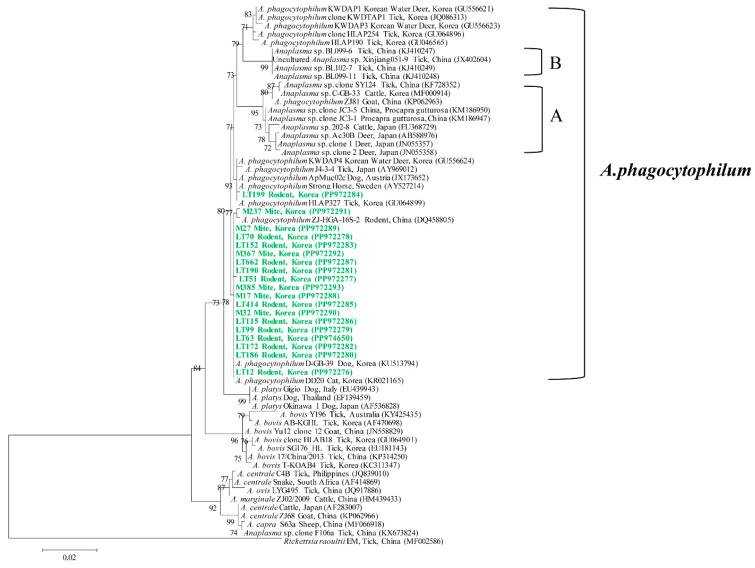
A phylogenetic tree was constructed from the 16s rRNA sequences of *Anaplasma* spp. using the maximum likelihood method. The sequences examined in this investigation are denoted in green. GenBank accession numbers for the referenced sequences are listed alongside their respective sequence names. *Rickettsia raoultii* was used as the outgroup. Bootstrap support values (based on 1000 replicates) are shown at the branches, and the scale bar denotes phylogenetic distance.

Nineteen representative phylogenetically analyzed samples and their 16s rRNA sequences were classified as *A. phagocytophilum* in both rodents and mites (Figure 1). The sequences exhibited a similarity range of 99.0–100% among themselves and displayed an identity of 98.1–100% with *A. phagocytophilum* isolates previously documented in GenBank. The sequences uncovered in this investigation have been deposited in GenBank and can be accessed via the accession numbers PP972276–PP972293 and PP974650.

**Figure 2:**
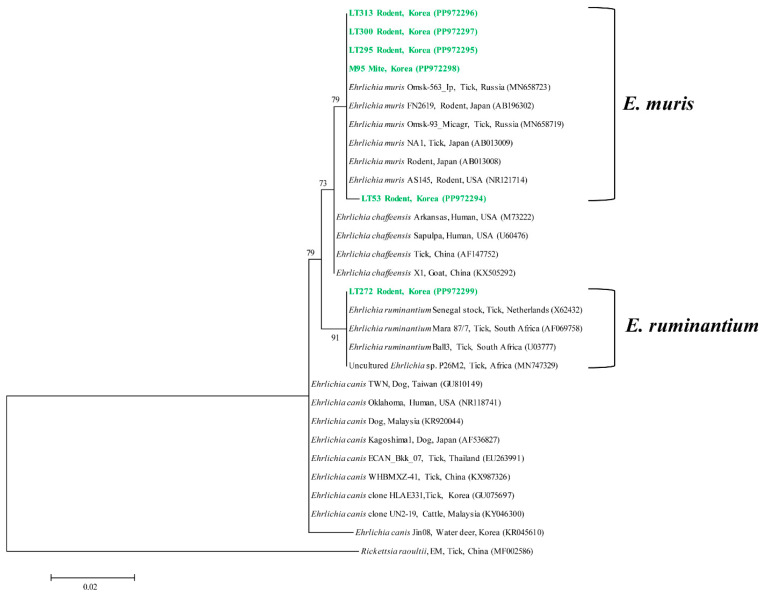
A phylogenetic tree was constructed from the 16s rRNA sequences of *Ehrlichia* spp. using the maximum likelihood method. The sequences investigated in this research are denoted in green, while GenBank accession numbers for the remaining sequences are listed adjacent to their respective sequence names. *Rickettsia raoultii* was used as the outgroup. Bootstrap support values (derived from 1000 replicates) are displayed at the branches, and the scale bar represents phylogenetic distance.

In the case of *Ehrlichia*, phylogenetic analysis revealed that our 16s rRNA sequences clustered as follows: five sequences were classified as *E. muris*, while one sequence was classified as *E. ruminantium* (Figure 2). The five sequences exhibited a similarity range of 99.7–100% among themselves and showed an identity of 99.4–100% with *E. muris* isolates previously documented in GenBank. These sequences have been deposited in GenBank and can be accessed via the accession numbers PP972294–PP972298. The *E. ruminantium* sequence displayed an identity of 100% with *E. ruminantium* isolates previously documented in GenBank and has also been submitted to GenBank, accessible via the same accession numbers: PP972299.

**Figure 3:**
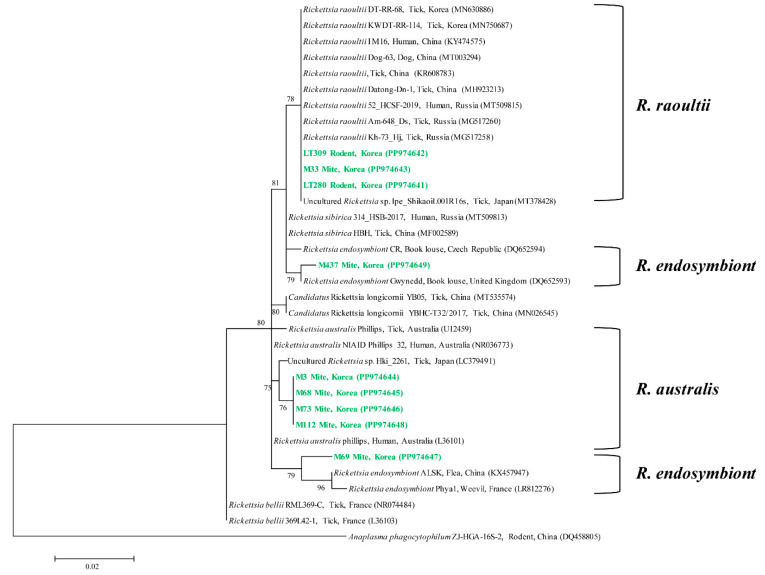
A phylogenetic tree was constructed from the 16s rRNA sequences of *Rickettsia* spp. using the maximum likelihood method. The sequences investigated in this study are denoted in green, while GenBank accession numbers for the remaining sequences are listed alongside their respective sequence names. *Anaplasma phagocytophilum* was used as the outgroup. Bootstrap support values (based on 1000 replicates) are shown at the branches, and the scale bar represents phylogenetic distance.

For *Rickettsia*, phylogenetic analysis revealed that three representative sequences were classified as *R. raoultii*, two sequences as *R. endosymbiont*, and four sequences as *R. australis* (Figure 3). The *R. raoultii* sequences exhibited a similarity range of 100% among themselves and an identity of 100% with previously documented *R. raoultii* isolates in GenBank. These sequences have been deposited in GenBank and can be accessed via the accession numbers PP974641–PP974643. The *R. endosymbiont* sequences exhibited a similarity range of 97.4% among themselves and an identity of 96.6–97.4% with previously documented *R. endosymbiont* isolates in GenBank. These sequences have also been deposited in GenBank and can be accessed via the same accession numbers: PP974647 and PP974649. Finally, the *R. australis* sequences exhibited a similarity range of 99.6–100% among themselves and an identity of 99.3–99.6% with previously documented *R. australis* isolates in GenBank. These sequences have been deposited in GenBank and can be accessed via the accession numbers PP974644–PP974646 and PP974648.

## 4. Discussion

Over the past two decades, global concern over tick-borne diseases has intensified due to emerging pathogens, raising significant public health concerns [19]. Surveillance programs for tick-borne diseases are crucial for understanding host–vector–pathogen interactions, which impact both animal and human populations [25]. Additionally, rodents act as reservoirs for various zoonotic pathogens, necessitating investigation into infections in both rodents and their vectors to understand the transmission of VBDs. In a 2020 study across 16 regions of Korea, *Ap. agrarius* comprised 76.4% of collected specimens, highlighting its predominance [4]. This study similarly found *Ap. agrarius* to be the most commonly encountered species in Korea. Mites collected from rodents in our study belong to the Trombiculidae family, which has a widespread distribution across Korea, with 15 genera and 63 species reported [4].

Ticks naturally transmit *Anaplasma* to numerous vertebrate hosts, including humans and animals [26]. *A. phagocytophilum* infections have been documented in various wild and domestic animals, particularly rodents [27]. The first suspected human case was reported in the USA in 1994 [28], and since then cases of human granulocytic anaplasmosis have increased [29]. It is important to differentiate between pathogenic *A. phagocytophilum* and closely related *A. phagocytophilum*-like species (APL), which are generally considered nonpathogenic and do not induce clinical symptoms in infected animals [30]. Previous molecular studies have identified various *Anaplasma* species across different hosts in Korea. For instance, *A. capra* and *A. bovis* have been detected in ticks parasitizing water deer (*Hydropotes inermis argyropus*) [31]; *A. capra*, *A. bovis*, APL clade A, and APL clade B in ticks from cattle [32]; *A. bovis* in ticks from native Korean goats [33]; *A. phagocytophilum*, APL clade B, *A. capra*, and *A. bovis* in questing ticks [34]; *A. capra* and *A. bovis* in cattle [35]; *A. phagocytophilum* and APL clade A in cattle [36]; *A. phagocytophilum* in dogs [37]; *A. phagocytophilum* in cats [38]; *A. phagocytophilum* and *A. bovis* in horses [39]; and *A. phagocytophilum* in humans and the ticks that have bitten them [40]. In China, the infection rate of *A. phagocytophilum* is significantly higher in summer due to the increased activity of primary vectors during this season [41]. Similarly, our study found a higher positivity rate of *A. phagocytophilum* in rodents and mites in southern regions, likely influenced by higher temperatures compared to other areas. In rodents, *A. phagocytophilum* was reported at 8.8% in China in 2006 [6] and 19.1% in Korea in 2016 [13]. In this study, *A. phagocytophilum* was detected in six out of ten rodent species, indicating that this pathogen is widely distributed in Korean rodents. Additionally, *A. phagocytophilum* was also detected in mites collected from three out of ten rodent species. To our knowledge, this represents the first detection of this pathogen in mites worldwide. Therefore, further epidemiological investigations on rodents and mites are crucial to deepen our understanding of the risks associated with other VBDs.

*Ehrlichia* species, obligate intracellular bacteria transmitted by ticks, are the causative agents of human monocytic ehrlichiosis [11]. *E. muris* was first isolated from a mouse in Japan in 1983 [42]. In Korea, 1.1% of ticks isolated from humans in 2020 tested positive for *Ehrlichia* sp. [17]. These cases suggest that *Ehrlichia* spp. not only uses animals as hosts but also humans, indicating its potential as a zoonotic disease. Studies have documented *E. muris* infections in various rodent species worldwide, including a 0.4% positivity rate in *Peromyscus leucopus* in the United States from 2014 to 2020 [7] and a 3.3% positivity rate in *Apodemus flavicollis* in Slovakia in 2006 [8]. In this study, *E. muris* was detected in *Ap. agrarius* and also found in mites collected from *Ap. agrarius*, marking the first domestic detection of *E. muris* in rodents and its presence in mites globally. These findings underscore the need for further investigation into the relationship between infections in rodents and mites, as well as the assessment of zoonotic infections and VBDs nationwide.

Wild animal species, particularly those affected by heartwater, a noncommunicable tick-borne ailment, are also impacted by *E. ruminantium*, which infects both domestic and wild ruminants [43]. In Africa, heartwater caused by *E. ruminantium* showed an incidence rate of 15% in cattle in 2014 [44], while in Uganda *E. ruminantium* was detected in 0.5% of goats in 2020 [45]. A 2016 case study in Mali found *E. ruminantium* in 33.3% (1/3) of *Amblyomma variegatum* collected from small mammals, suggesting that various small mammal species may act as reservoirs for zoonotic infections [46]. Previously, *E. ruminantium* was typically prevalent in ruminants; however, in this study it was found in rodents, representing the first discovery of *E. ruminantium* in rodents worldwide. *E. ruminantium* was not detected in mites in our study. Although *E. ruminantium* is not zoonotic, given the increasing global trend of various VBDs, establishing monitoring and surveillance systems for these pathogens is necessary.

Rickettsiae are transmitted by various arthropods, such as fleas, mites, ticks, and lice, which serve as both reservoirs and vectors for infecting humans and animals [19,47]. Diseases caused by the spotted fever group Rickettsiae affect wildlife, humans, and domesticated animals [48]. For instance, in a German study conducted from 2006 to 2008, *R. raoultii* was found in 30.3% of ticks, while none of the 119 rodents tested positive for *Rickettsia* spp. [49]. In Kazakhstan, 2.72% of rodents tested positive for *R. raoultii* between 2018 and 2019 [9]. In Spain, 3.7% of blood samples from patients collected between 1990 and 2004 tested positive for *R. raoultii* [50]. In Korea, *R. raoultii* was detected in 40.9% of dog ticks from 2010 to 2015 [51]. Conversely, a Korean study from 2004 to 2005 sampled 369 rodents, including *Ap. agrarius*, and found no *Rickettsia* spp. [12]. However, in this study, *R. raoultii* was found in both *Ap. agrarius* and *Crocidura* spp. Notably, among them only *R. raoultii* was found to infect *Crocidura* spp. in addition to *Ap. agrarius*, the dominant species in Korea. These findings can expand our understanding of rodent species in Korea that may contribute to the occurrence of zoonotic diseases. The novel detection of *R. raoultii* in rodents in Korea, as well as in mites worldwide, underscores their potential as disease vectors to humans. This necessitates continued assessment of the public health risks posed by these infected animals.

Symbiotic microorganisms, including intracellular *Rickettsia*, are prevalent in most insects and influence their traits. In Rickettsiae phylogeny, hard ticks (*Ixodidae*) consistently appear, suggesting ticks were the original hosts for Rickettsial species associated with arthropods. Over time, some *Rickettsia* species have adapted to other eukaryotes, such as protozoa and leeches [52]. From 2011 to 2013, a study in Slovakia analyzed 991 rodents, detecting *R. endosymbionts* in two individuals, resulting in a prevalence rate of 0.2% [10]. In a study conducted in China from 2014 to 2016, 0.04% of the collected fleas tested positive for two species of *Rickettsia* spp., one of which was identified as an *R. endosymbiont* [53]. In collections of spider mites from southwestern Europe (Portugal, Spain, France) in 2013, the prevalence of *R. endosymbiont* was found to be 2.7% [54]. While it may be challenging to compare spider mites (family: Tetranychidae) with mites collected from rodents in our study (family: Trombiculidae), the presence of endosymbionts is still significant and similar to our findings. In this study, *R. endosymbionts* were not detected in rodents but were found in mites, marking the first discovery of *R. endosymbionts* in mites worldwide. Therefore, additional research is needed on the interactions and roles between vectors and animals for *Rickettsia*.

The pathogen responsible for Queensland tick typhus (QTT), *R. australis*, is increasingly being recognized as a significant cause of acute febrile illness in eastern Australia [55]. QTT was first documented in Queensland in 1946, with subsequent cases reported in New South Wales and Victoria [56]. In Australia, QTT was observed in 36 of 50 suspected human cases in hospitals from 2000 to 2015 [57], and 21.4% of individuals working as tick collectors had antibodies to *R. australis* [58]. These cases suggest that *R. australis* can infect humans through ticks. Outside of Australia, a study conducted in Japan from 2013 to 2016 detected R. australis in 4.1% of ticks [59]. Given that *R. australis* is relatively rare in Australia [55], there has not been much research conducted on it. However, its discovery in Japan, which is close to Korea, suggests that Asia cannot be considered safe from this pathogen. In our research, *R. australis* was detected in mites, marking the first documented cases of *R. australis* detection in mites worldwide. Additionally, this is the only confirmed case of infection found in mites collected from *M. minutus* and *Ap. agrarius*. This suggests that the disease may also occur in other rodent species, not just in the dominant *Ap. agrarius* in Korea. Notably, in this study *Ehrlichia* and *Rickettsia* were primarily detected in the northern regions rather than the southern regions. This contrasts with the common trend of zoonotic diseases occurring mainly in the southern regions of Korea, where higher average temperatures tend to correlate with increased positivity rates [41,60]. These findings significantly expand the known geographical distribution of the pathogens and highlight the need for ongoing surveillance of humans, animals, and vectors.

Phylogenetic analysis demonstrated that *A. phagocytophilum* sequences from rodents and mites in this study share a high identity with sequences from a range of hosts in Korea, including Korean water deer, cattle, ticks, dogs, and cats. Notably, they also showed a strong similarity to sequences from rodents in China (DQ458805) and ticks in Japan (AY969012), which highlights the close genetic relationship and suggests the potential for cross-border transmission of pathogens. Additionally, it is important to note that *A. phagocytophilum* is not the only species of *Anaplasma* prevalent in Korea and globally. Other species, such as *A. bovis, A. capra*, and APL clades A and B, have been detected in ticks from Korea, further emphasizing the need for comprehensive surveillance.

Similarly, the *E. muris* sequences from rodents and mites shared a high identity with sequences from rodents in the USA (NR121714) and Japan (AB013008), as well as with those detected in ticks. The *E. ruminantium* sequence from rodents showed a strong similarity to sequences found in ticks. In addition to *E. muris* and *E. ruminantium*, *E. chaffeensis* and *E. canis* have been identified in ticks and humans, raising concerns about the potential zoonotic transmission of *Ehrlichia* species between ticks and humans.

*R. raoultii* sequences from rodents and mites in this study exhibited a strong identity with sequences from humans, ticks, and dogs. Notably, *R. endosymbiont* sequences from mites showed a 96.6–97.4% identity with sequences from book lice, fleas, and weevils. The *R. australis* sequences from mites displayed a high identity with sequences from ticks and humans in Australia, as well as ticks in Japan.

While *R. endosymbiont* and *R. australis* are relatively less studied compared to *R. raoultii*, the detection of *R. endosymbiont* in various arthropods, including ticks and mites, underscores the need for further sequence analysis across different arthropod species. Similarly, *R. australis* has been found in humans, mites, and ticks, indicating that more research is required to better understand its transmission dynamics and potential for wider dissemination.

## 5. Conclusions

Overall, this research conducted an extensive nationwide survey on the regional distribution of rodents and mites, combined with molecular analyses of three VBDs. This study reported the first domestic cases of *E. muris* and *R. raoultii* in rodents. It also marked the first global detection of *A. phagocytophilum*, *E. muris*, *R. raoultii*, *R. endosymbiont*, and *R. australis* in mites, as well as the first global detection of *E. ruminantium* in rodents. These findings underscore the risk of zoonotic diseases carried by rodents and mites, emphasizing the need for public awareness during outdoor activities. Further geographical and ecological studies on these vectors will enhance our understanding of the risk of these diseases in Korea. Therefore, additional research is necessary to examine the distribution of rodents and mites to better comprehend the epidemiology of VBDs and their public health risks in Korea.

## Figures and Tables

**Table 1:** Number of rodents and mites collected and infected with VBDs in Korea.

Species	No. of Collected		No. of Infected (%)											
	Rodents	Mites (Pools)	*A. phagocytophilum*		*E. muris*		*E. ruminantium*		*R. raoultii*		*R. endosymbiont*		*R. australis*	
			Rodents	Mite Pools	Rodents	Mite Pools	Rodents	Mite Pools	Rodents	Mite Pools	Rodents	Mite Pools	Rodents	Mite Pools
*Apodemus agrarius*	693	7491 (454)	73 (10.5)	42 (9.3)	23 (3.3)	1 (0.2)	2 (0.3)	0	21 (3.0)	1 (0.2)	0	8 (1.8)	0	5 (1.1)
*Apodemus peninsulae*	5	57 (4)	2 (40.0)	1 (25.0)	0	0	0	0	0	0	0	0	0	0
*Craseomys regulus*	13	125 (9)	1 (7.7)	1 (11.1)	0	0	0	0	0	0	0	0	0	0
*Craseomys rufocanus*	1	4 (1)	1 (100)	0	0	0	0	0	0	0	0	0	0	0
*Crocidura lasiura dobson*	1	0	0	0	0	0	0	0	0	0	0	0	0	0
*Crocidura* spp.	86	50 (9)	8 (9.3)	0	0	0	0	0	3 (3.5)	0	0	0	0	0
*Micromys minutus*	25	104 (10)	1 (4.0)	0	0	0	0	0	0	0	0	0	0	1 (10)
*Microtus fortis*	4	94 (4)	0	0	0	0	0	0	0	0	0	0	0	0
*Mogera robusta*	1	18 (1)	0	0	0	0	0	0	0	0	0	0	0	0
*Myodes regulus*	6	24 (6)	0	0	0	0	0	0	0	0	0	0	0	0
Total	835	7971 (498)	86 (10.3)	44 (8.8)	23 (2.8)	1 (0.2)	2 (0.2)	0	24 (2.9)	1 (0.2)	0	8 (1.6)	0	6 (1.2)

*A.*, *Anaplasma*; *E.*, *Ehrlichia*; *R.*, *Rickettsia*. Statistical analysis showed no significant differences for the rodent or mite data.

**Table 2:** Data on the regional and seasonal distribution of VBDs and infection rates in rodent and mite samples in Korea.

Group			No. of Tested				No. of Infected (%)									
			Rodents	Mites (Pools)	*A. phagocytophilum*		*E. muris*		*E. ruminantium*		*R. raoultii*		*R. endosymbiont*		*R. australis*	
					Rodents	Mite Pools	Rodents	Mite Pools	Rodents	Mite Pools	Rodents	Mite Pools	Rodents	Mite Pools	Rodents	Mite Pools
Regions	Northern	Cheorwon	87	944 (53)	8 (9.2)	7 (13.2)	5 (5.7)	0	2 (2.3)	0	9 (10.3)	0	0	0	0	1 (1.9)
		Gangneung	76	426 (34)	4 (5.3)	2 (5.9)	15 (19.7) *	0	0	0	12 (15.8) *	0	0	0	0	0
		Yeoju	78	736 (57)	16 (20.5)	3 (5.3)	3 (3.8)	0	0	0	3 (3.8)	1 (1.8)	0	0	0	0
		Hwasung	45	635 (40)	1 (2.2)	2 (5.0)	0	0	0	0	0	0	0	4 (10.0) *	0	0
		Paju	56	984 (46)	5 (8.9)	2 (4.3)	0	0	0	0	0	0	0	3 (6.5)	0	2 (4.3)
	Central	Cheongju	36	489 (23)	5 (13.9)	2 (8.7)	0	0	0	0	0	0	0	0	0	0
		Boryeong	35	279 (16)	1 (2.9)	1 (6.3)	0	0	0	0	0	0	0	0	0	0
		Yesan	33	426 (19)	2 (6.1)	0	0	0	0	0	0	0	0	0	0	1 (5.3)
	Southern	Jeongeup	43	751 (39)	8 (18.6)	7 (17.9)	0	0	0	0	0	0	0	0	0	2 (5.1)
		Boseong	41	443 (32)	6 (14.6)	2 (6.3)	0	1 (3.1)	0	0	0	0	0	0	0	0
		Jinan	49	404 (35)	5 (10.2)	0	0	0	0	0	0	0	0	0	0	0
		Geoje	41	318 (14)	18 (43.9) *	1 (7.1)	0	0	0	0	0	0	0	0	0	0
		Hapcheon	36	98 (12)	2 (5.6)	3 (25.0)	0	0	0	0	0	0	0	0	0	0
		Gimcheon	28	296 (17)	0	1 (5.9)	0	0	0	0	0	0	0	1 (5.9)	0	0
		Yeongdeok	47	522 (41)	5 (10.6)	11 (26.8) *	0	0	0	0	0	0	0	0	0	0
	Jeju Island	Seogwipo	104	220 (20)	0	0	0	0	0	0	0	0	0	0	0	0
Seasons	Spring		560	3734 (299)	85 (15.2) *	43 (14.4) *	1 (0.2)	1 (0.3)	0	0	0	1 (0.3)	0	8 (2.7) *	0	6 (2.0)
	Autumn		275	4237 (199)	1 (0.4)	1 (0.5)	22 (8.0) *	0	2 (0.7) *	0	24 (8.7) *	0	0	0	0	0
Total			835	7971 (498)	86 (10.3)	44 (8.8)	23 (2.8)	1 (0.2)	2 (0.2)	0	24 (2.9)	1 (0.2)	0	8 (1.6)	0	6 (1.2)

*A.*, *Anaplasma*; *E.*, *Ehrlichia*; *R.*, *Rickettsia*. * Significant differences in prevalence (*p* < 0.05).

## Data Availability

Data supporting the conclusions of this article are included within this article. The newly generated sequences were submitted to the GenBank database under the accession numbers PP972276–PP972299 and PP974641–PP974650. The datasets used and/or analyzed during the present study are available from the corresponding author upon reasonable request.
